# Characterizing Ethnomedicinal *Tetrastigma hemsleyanum* Diels et Gilg Grown Under Different Cultivation Methods Using Stable Isotopes and Elemental Analyses

**DOI:** 10.3390/plants15101589

**Published:** 2026-05-21

**Authors:** Chunan Wang, Xianbo Wang, Hanyi Mei, Yongzhi Zhang, Chunlin Li, Karyne M. Rogers, Zuguang Li, Yuwei Yuan, Jing Nie

**Affiliations:** 1 College of Chemical Engineering, Zhejiang University of Technology, Hangzhou 310014, China; 221123010446@zjut.edu.cn; 2 State Key Laboratory for Quality and Safety of Agro-Products, Zhejiang Academy of Agricultural Sciences, Hangzhou 310021, China; mhyaqq@163.com (H.M.); zhangyz@zaas.ac.cn (Y.Z.); chunlinli0304@163.com (C.L.); karynerogers@gmail.com (K.M.R.); yuanyw@zaas.ac.cn (Y.Y.); 3 Key Laboratory of Information Traceability for Agricultural Products, Ministry of Agriculture and Rural Affairs of China, Institute of Agro-Products Safety and Nutrition, Zhejiang Academy of Agricultural Sciences, Hangzhou 310021, China; 4 Hangzhou Academy of Agricultural Sciences, Hangzhou 310024, China; chayesuo2008@163.com; 5 Department of Soil and Physical Sciences, Lincoln University, Lincoln 7647, New Zealand

**Keywords:** *Tetrastigma hemsleyanum*, stable isotopes, metals, agro-forestry system

## Abstract

*Tetrastigma hemsleyanum* Diels et Gilg is a high-value edible and medicinal homologous plant, routinely grown under conventional field or greenhouse production systems across Asia. However, mislabeling of conventional products as the rarer (and more expensive) wild version may occur for financial gain. In this study, stable isotopes (*δ*^13^C, *δ*^15^N, *δ*^2^H, and *δ*^18^O) and metal contents (Cr, Cu, Ni, As, Cd, Pb) were used to characterize plant tissues (tuber root, stem, leaf) and corresponding soils originating from simulated-wild-cultivated (WC) and greenhouse-cultivated (GC) pot trials using the same soil. Carbon and nitrogen isotopes served as key indicators for distinguishing GC and WC products. Specifically, *δ*^13^C values of GC plant tissues were 1.4 to 2.4‰ more positive than those of WC plant tissues (*p* < 0.05), and *δ*^15^N values in GC tissues were 2.7 to 4.6‰ more positive than *δ*^15^N in WC tissues (*p* < 0.01). Lower *δ*^15^N values observed in WC products indicate slower nitrogen turnover compared with GC products. Soil metal concentrations had significant differences between the two cultivation systems, but only limited effects on metal bioconcentration factors (BCFs) and translocation factors (TFs) in *T. hemsleyanum* tissues. Pb and Cd concentrations in root tissues had large differences between cultivation systems, and carbon dynamics in GC plants were more negatively affected by Pb levels in soils. These findings provide the first investigation of *T. hemsleyanum* grown under different cultivation practices and establish a scientific basis for distinguishing other wild or simulated-wild labeled food and medicinal plant products from conventionally grown products in future studies.

## 1. Introduction

*Tetrastigma hemsleyanum* Diels et Gilg (*T. hemsleyanum*), also known as “San Ye Qing” or *T. hemsleyanum*, is a renowned edible and medicinal herb in Asia and is widely used to formulate different dietary supplements. The leaves are commonly consumed as a functional or dietary supplement in China, while the tuber roots are processed through boiling, squeezing or extraction for the preparation of soups, juices and nutraceutical formulations [1]. It is predominantly distributed in mountainous regions of southern China, as well as in India and Southeast Asian countries such as Vietnam, Myanmar, and Thailand. The plant from Zhejiang Province, China, has a slower growth cycle than that from other locations and consequently a higher quality and market value.

Agro-forestry (or simulated-wild cultivation) production methods have become increasingly popular over the past few years, as they provide healthier soil, greater biodiversity, and higher quality food production [2]. Increasing attention is now paid to the development of this sustainable farming system, which has a lower reliance on agro-chemicals and fossil fuels and aims to ensure environmental preservation and food security [3]. *T. hemsleyanum* products sold into traditional medicine markets are labeled as wild, simulated-wild, and greenhouse-cultivated, and are sold at higher prices when produced under natural (wild) or simulated-wild conditions, thus creating a need to differentiate between growing systems. Generally, *T. hemsleyanum* is cultivated using agro-forestry systems (wild or simulated-wild products) and has a higher product quality than conventionally grown counterparts [4]. However, there are very few studies that have determined agricultural system differences and environmental uptake of soil metals from agro-forestry and greenhouse cultivation of medicinal plants. Therefore, the opportunity to study *T. hemsleyanum* under these different production systems offers new insights regarding the differentiation of agro-forestry plants from conventionally farmed products.

Stable isotope and multi-elemental techniques exhibit significant correlations with plant growth processes and have been used to authenticate and verify different growing methods [5]. Organic farming (using organic fertilizers) has significant effects on plant stable isotopes. Bontempo et al. [6] studied tomatoes grown using different fertilizers and noted that the nitrogen isotope (*δ*^15^N) values of tomatoes grown under organic conditions were more positive than those under conventional methods. Natural or organic Huangjing (*Polygonati Rhizoma*), a high-value medicinal and food homology, showed significantly higher mean *δ*^15^N values (6.8‰) than conventionally farmed products (0.7‰), although mean carbon isotope (*δ*^13^C) values were generally similar at around −28.0‰ [7].

Organic fertilizers have also been shown to influence rice carbon isotopes, as ^12^CO_2_ released from microbial decomposition of organic fertilizers is absorbed by plants, resulting in more negative *δ*^13^C values in organic rice [8]. Furthermore, more positive *δ*^18^O values and lower *δ*^15^N values were observed in wild *Camellia sinensis* samples (25.7‰ and 2.3‰, respectively) [9]. Lower *δ*^15^N values in wild plant resources are often linked to biological nitrogen (N) fixation, atmospheric N deposition and low soil disturbance, while *δ*^15^N values of plants using organic fertilizers such as animal manures have more positive values. Microbial denitrification of organic manures applied to soil results in the preferential removal of the lighter ^14^N isotope, enriching the residual soil ^15^N during mineralization and its subsequent uptake by plants [10]. Hydrogen and oxygen (*δ*^2^H and *δ*^18^O) isotope values of *Platycladus orientails*, *Prunus davidiana* and *Medicago sativa* plants and their soil water were compared to develop a model to evaluate water uptake efficiency in an agro-forestry system in drought regions [11].

Metal elements are indispensable for plant physiology and are sourced from soils and fertilizers. Some metals, such as copper (Cu) and nickel (Ni), play a significant role in plant growth and development. Cu is a transition metal that contributes to the biosynthesis of alkaloids, flavonoids, lignans and other aromatic compounds in plants [12,13]. Ni is an essential plant nutrient and is critical for nitrogen metabolism, acting as an active catalyst for urease and a co-factor in the formation of specific superoxide dismutase isoenzymes [14]. However, medicinal herbs may accumulate some toxic or non-essential metals during cultivation, highlighting ecosystem health risks. Excessive metal uptake of As, Cd, Cr, and Pb causes negative impacts on plant growth and metabolism. Toxic metals not only interfere with diverse plant metabolic processes—including water and nutrient uptake, translocation, photosynthesis, and respiration—but also impose persistent and long-term impacts on the environment [15,16,17].

Plant metal uptake from soils, as well as their accumulation and translocation from roots to above-ground tissues, is complex, and studies reporting cultivation type influences are limited. Moreover, carbon and nitrogen cycling may be affected by higher toxic metal concentrations in different ecosystems or agricultural production systems. Previous research has shown an increase in carbon and nitrogen stable isotope values of two castor plant species under different Cd treatments [18]. *δ*^15^N values have also shown positive correlations with Cd, Cu, and Pb in soils impacted by industrial/municipal wastewater, while *δ*^13^C did not consistently reflect changes with metal contamination [19]. Under different plant cultivation systems, metal distributions have been shown to affect carbon or nitrogen flux, assimilation pathways, as well as metal allocations in soil-plant systems.

This study focuses on characterizing stable isotopes and metals in different plant tissues (leaves, stems, and roots) from *T. hemsleyanum,* grown using two cultivation methods: greenhouse cultivation (GC) and simulated-wild cultivation (WC). Additionally, by integrating soil isotopes (*δ*^13^C and *δ*^15^N values) and elemental data from the two cultivation systems, the effect of elemental uptake, bioaccumulation and translocation in *T. hemsleyanum* is examined. Furthermore, relationships between soil/plant metal distributions and carbon and nitrogen isotopic compositions (*δ*^13^C and *δ*^15^N) are explored to verify farming differences. This research supports the development of *T. hemsleyanum* from different cultivation pathways and establishes a resource protection technology system, which provides more comprehensive and theoretical information for future quality enhancement of *T. hemsleyanum* and other medicinal food plants.

## 2. Results and Discussion

### 2.1. Tissue Stable Isotope Differences Between Cultivation Methods

Stable isotope (*δ*^13^C, *δ*^15^N, *δ*^2^H, and *δ*^18^O) values of three *T. hemsleyanum* tissue types (leaves, stems and roots) and their fractionation characteristics under the two cultivation methods are shown in Table 1. Notably, the *δ*^13^C and *δ*^15^N values exhibited significant variations between cultivation methods.

Mean *δ*^13^C values of GC *T. hemsleyanum* were −30.4 ± 1.3‰ (leaves), −30.1 ± 1.0‰ (stems), and −28.3 ± 1.3‰ (roots), whereas WC plants were −31.8 ± 1.0‰ (leaves), −32.1 ± 1.4‰ (stems), and −30.7 ± 1.5‰ (roots). The *δ*^13^C values of the WC plant tissues were significantly lower than those of their GC counterparts (*p* < 0.05). Mean *δ*^13^C values of GC leaves were more negative than those of GC stems, with a stem–leaf *α* of 0.9997. Mean *δ*^13^C values of WC leaves were more positive than those of WC stems, with a stem–leaf α of 1.0003. These results suggest that under greenhouse cultivation, *T. hemsleyanum* leaves experience lower water stress and higher stomatal conductance, leading to greater within-plant discrimination against ^13^C (more negative leaf *δ*^13^C values), whereas under simulated-wild cultivation, plants likely suffer from water deficits or higher light intensity, causing partial stomatal closure, reducing CO_2_ supply, and resulting in more positive leaf *δ*^13^C values relative to their stems [20]. *δ*^13^C differences in plants are frequently attributed to CO_2_ fractionation during photosynthesis, caused by light, heat and water stress that result in stomatal closure. Simulated-wild cultivated *T. hemsleyanum* typically grows under forest canopies with higher shading and cooler temperatures than greenhouse-cultivated plants, leading to more negative *δ*^13^C tissue values than corresponding greenhouse specimens.

GC *T. hemsleyanum* exhibited higher *δ*^15^N values across all tissue types—leaves (6.0 ± 1.4‰), stems (4.6 ± 2.1‰), and roots (4.1 ± 1.2‰)—than in WC plants—roots (1.4 ± 2.3‰), stems (0.3 ± 1.4‰), and leaves (1.4 ± 1.8‰) (*p* < 0.05)—with α mostly >1 in both systems. During root–stem ^15^N fractionation, the WC samples displayed lower root–stem *α* values (0.9989) than did the GC samples (1.0005), while for stem–leaf ^15^N fractionation, the GC and WC samples exhibited similar patterns with, an α of 1.0012, indicating that water/nutrient stress under WC conditions altered nitrogen transport/assimilation in roots and stems but in leaf-level processes. Plant *δ*^15^N values are known to be influenced by fertilizer type and application amount during growth, and the application of organic fertilizers can significantly increase plant *δ*^15^N values compared to unfertilized or chemically fertilized plants. However, given that both GC and WC cultivation methods received the same organic sheep manure pellets, key *δ*^15^N differences in plant tissues are most likely due to higher denitrification rates from more active microbial activity under warmer greenhouse conditions [21].

In contrast, there were no significant *δ*^18^O differences observed between the two cultivation systems (*p* = 0.441), with mean GC *T. hemsleyanum δ*^18^O values of 26.7 ± 1.8‰ (leaves), 20.9 ± 0.8‰ (stems), and 27.1 ± 0.9‰ (roots), compared to 27.2 ± 0.6‰ (leaves), 21.0 ± 0.8‰ (stems), and 26.4 ± 0.9‰ (roots) for WC plants. However, significant *δ*^18^O differences were detected in roots and leaves compared to the stems (*p* < 0.05). The *δ*^18^O root–stem α values were <1, but the stem–leaf α values were >1, indicating that leaves and roots are the main organs involved in oxygen transportation [22]. Photosynthesis and evapotranspiration are primary drivers of *δ*^18^O variations in plant tissues. Notably, the stem functions as the main conduit for water transportation from roots to leaves, so it does not experience the same amount of fractionation as leaves and roots. Photosynthesis and stomatal closure actively affect the *δ*^18^O values in plant leaves, and soil–plant–bacterial oxygen exchange affects the root *δ*^18^O values, with the GC samples having wider variations in *δ*^18^O values due to larger diurnal temperature fluctuations in the greenhouse than those experienced in the forest by WC plants [23].

There were no significant *δ*^2^H differences observed between the two cultivation systems, although GC specimens exhibited more negative mean *δ*^2^H values across all tissue types: −65 ± 7‰ (leaves), −59 ± 4‰ (stems), and −13 ± 5‰ (roots), compared to WC plants: −62 ± 10‰ (leaves), −50 ± 6‰ (stems), and −12 ± 5‰ (roots), with significant *δ*^2^H tissue differences found in stems (*p* < 0.05). Similar to *δ*^18^O values, water availability and evapotranspiration are primary drivers of *δ*^2^H variations in plants [23]. Under greenhouse cultivation, regular irrigation and elevated daytime temperatures intensify transpiration and fractionation processes, leading to lower *δ*^2^H values observed in greenhouse-cultivated *T. hemsleyanum*. Roots had more positive *δ*^2^H values than above-ground tissues, with a root–stem α of 0.9534 and 0.9615 in GC and WC samples, respectively. Stems and leaves underwent progressive ^2^H depletion compared to the roots, and displayed slightly lower stem-leaf α values of 0.9936 and 0.9874 in GC and WC samples [24]. These findings indicate that roots act as ^2^H-enriched reservoirs within the plant, with ^2^H depletion occurring during upward translocation across aerial tissues. The root *δ*^2^H values are more enriched in ^2^H than the stems and leaves due to conduit fractionation, which enhances the more mobile ^1^H isotope in distal tissues [23].

### 2.2. Tissue-Specific Stable Isotope Relationships

**Figure 1:**
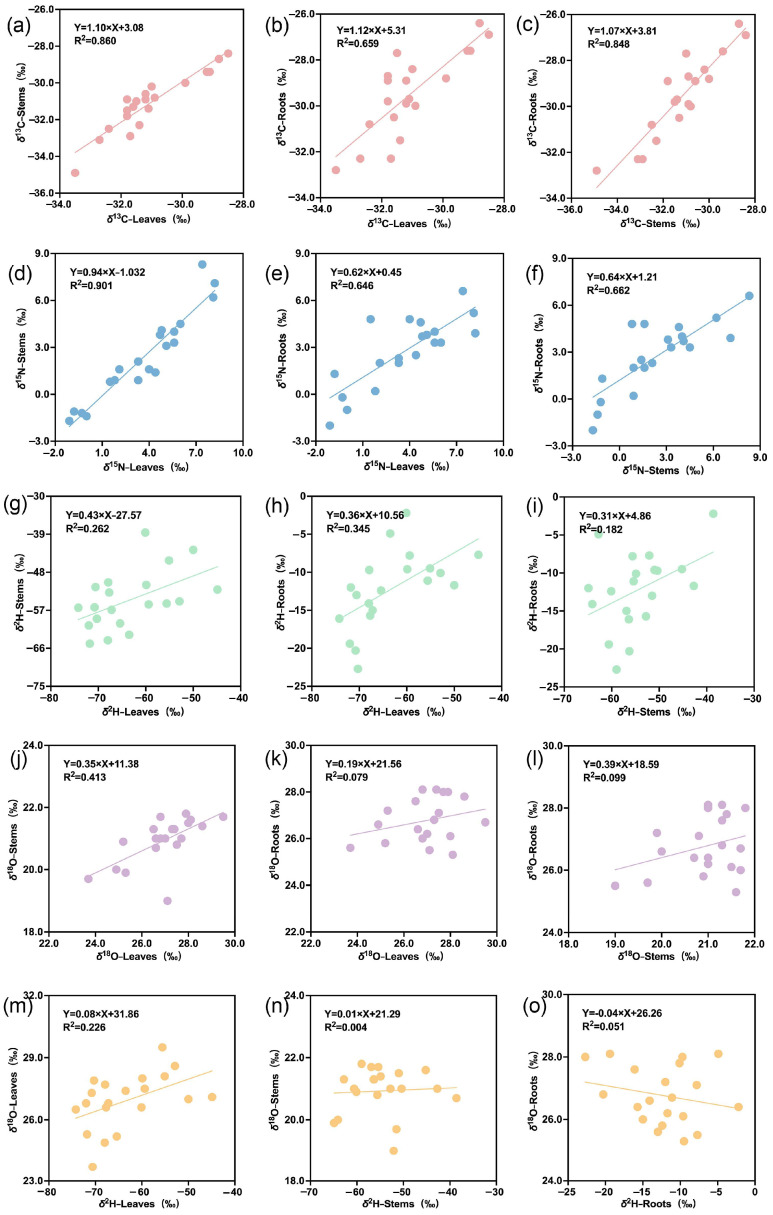
Stable isotope correlations between leaf, stem, and root tissues. (**a**) δ^13^C between leaves and stems; (**b**) δ^13^C between leaves and roots; (**c**) δ^13^C between stems and roots; (**d**) δ^15^N between leaves and stems; (**e**) δ^15^N between leaves and roots; (**f**) δ^15^N between stems and roots; (**g**) δ^2^H between leaves and stems; (**h**) δ^2^H between leaves and roots; (**i**) δ^2^H between stems and roots; (**j**) δ^18^O between leaves and stems; (**k**) δ^18^O between leaves and roots; (**l**) δ^18^O between stems and roots; (**m**) δ^2^H-δ^18^O between leaves; (**n**) δ^2^H-δ^18^O between stems; (**o**) δ^2^H-δ^18^O between roots.

The stable isotope correlation characteristics of different *T. hemsleyanum* tissue samples and cultivation methods are shown in Figure 1. Notably, *δ*^13^C values were found to be more positive in roots compared to above-ground tissues, aligning with other plant studies, and indicate physiological differences causing fractionation among tissues. More positive *δ*^13^C values are generally seen in non-respiring/non-photosynthetic tissues relative to photosynthetic tissues [25]. The *δ*^13^C values of different *T. hemsleyanum* tissue samples showed a strong linear correlation (*r* > 0.821, *p* < 0.01), and those between adjacent tissues (root–stem or stem–leaf) showed high regression coefficients (R^2^ > 0.848). Mean *δ*^15^N values of leaves were more positive than stem samples (*δ*^15^N_(leaf)_ > *δ*^15^N_(stem)_), and *δ*^15^N plant tissue values were well correlated (*r* > 0.646, *p* < 0.01), although the regression coefficient between stems and leaves (R^2^ = 0.901) was much higher than that between roots and stems (R^2^ = 0.662) and roots and leaves (R^2^ = 0.646). The *δ*^15^N values across different plant organs are a consequence of nitrogen uptake, loss, assimilation and translocation processes. Agro-forestry (simulated-wild) cultivation methods are subject to more natural (irregular) climate conditions, which are solely dependent on local weather patterns, in contrast to regularly irrigated and temperate conventional cropping systems; thus, nutrient and irrigation uptake cannot be easily predicted [26]. However, this study clearly shows a slower nitrogen turnover rate under WC conditions, as indicated by the lower *δ*^15^N values across WC tissues compared to GC tissues [27,28].

Furthermore, the GC and WC plant tissue *δ*^13^C and *δ*^15^N compositions revealed various plant physiological processes that comprehensively reflect different growth strategies and environmental adaptability under the two cultivation modes. GC plant tissues had more positive *δ*^13^C and *δ*^15^N values and higher isotopic correlations than WC tissues, suggesting that nutrient allocation and metabolism may be more regulated under GC conditions.

*δ*^2^H values exhibited significant differences among plant tissues: *δ*^2^H_(leaf)_ < *δ*^2^H_(stem)_ < *δ*^2^H_(root)_. The *δ*^2^H correlation between leaves and roots had a significantly higher coefficient (*r* = 0.587, *p* < 0.01) than those between leaves and stems (*r* = 0.512, *p* < 0.05) and stems and roots (*r* = 0.426, *p* > 0.05), as these tissues exhibited the largest fractionation differences.

*δ*^18^O values also exhibited significant differences among plant tissues: *δ*^18^O_(stem)_ < *δ*^18^O_(leaf)_ = *δ*^18^O_(root)_. Only leaves and stems had significant *δ*^18^O correlations (*r* = 0.643, *p* < 0.01), reflecting their shared physiological processes [29]. Irrigation water serves as a major source of oxygen in plants, and water transport within plants and CO_2_ respiration/transpiration processes both affect oxygen isotope fractionation across different plant tissues [30]. Moreover, the correlations between *δ*^2^H and *δ*^18^O values in each tissue type were explored, and the results showed that leaves had slightly higher correlations (*r* = 0.475, *p* < 0.01) than other tissues.

### 2.3. Effect of Cultivation Method on Soil Isotopes and Associated Plant Correlations

Soil carbon and nitrogen cycles are intertwined with plant growth. Soil carbon and nitrogen contents and their isotopes are shown in Table 2. %C and %N showed significant differences between the two cultivation systems, while *δ*^13^C and *δ*^15^N exhibited no significant differences. The application of organic fertilizers under GC conditions resulted in significantly higher %C and %N contents in GC soils than in similarly fertilized WC soils (*p* < 0.05). Plant organic detritus and exogenous fertilizers served as the primary input source of soil carbon and nitrogen. Nitrogen is further transformed to become the core supply pathway for plant nitrogen nutrition. The sheep manure organic fertilizer and residual soil organic matter (SOM) were the primary *δ*^13^C sources for both soils, which resulted in no significant difference in *δ*^13^C values between the two soils.

Choi et al. [31] noted in their global analysis of soil *δ*^15^N values that forest ecosystems were characterized by closed nitrogen cycles, low soil N turnover and limited ^15^N availability, and found lower soil *δ*^15^N values in forests than in farmlands and grasslands. Specifically, undisturbed subtropical forest soils had an average *δ*^15^N value of 2.9 ± 0.3‰, whereas actively cultivated soils with animal manures typically had a mean *δ*^15^N value of 7.8 ± 0.6‰. Manure application significantly alters soil *δ*^15^N values and subsequently influences plant tissue *δ*^15^N values. Notably, the soils used in both cultivation methods in this study originated from the same source, with the same sheep pellet fertilizer additions. Therefore, the higher *δ*^15^N values observed in GC *T. hemsleyanum* can only be attributed to climatic differences during cultivation, as the same fertilizers were applied to both GC and WC plants.

While manure-based organic fertilizers can elevate soil *δ*^15^N values, the higher ambient temperatures in greenhouses enhance microbial activity, which in turn intensifies soil nitrogen mineralization and nitrification [32]. This bacterial process promotes the conversion of animal waste (as urea or ammonia) into NH_4_^+^ and NO_3_^−^ and provides mineralized organic nitrogen that can be directly absorbed by plants. Consequently, GC *T. hemsleyanum* proportionally takes up more ^15^N nitrogen than WC plants, leading to a greater range of *δ*^15^N values compared to WC plants due to enhanced microbially driven denitrification and remineralization processes in the greenhouse cultivation soil.

**Figure 2:**
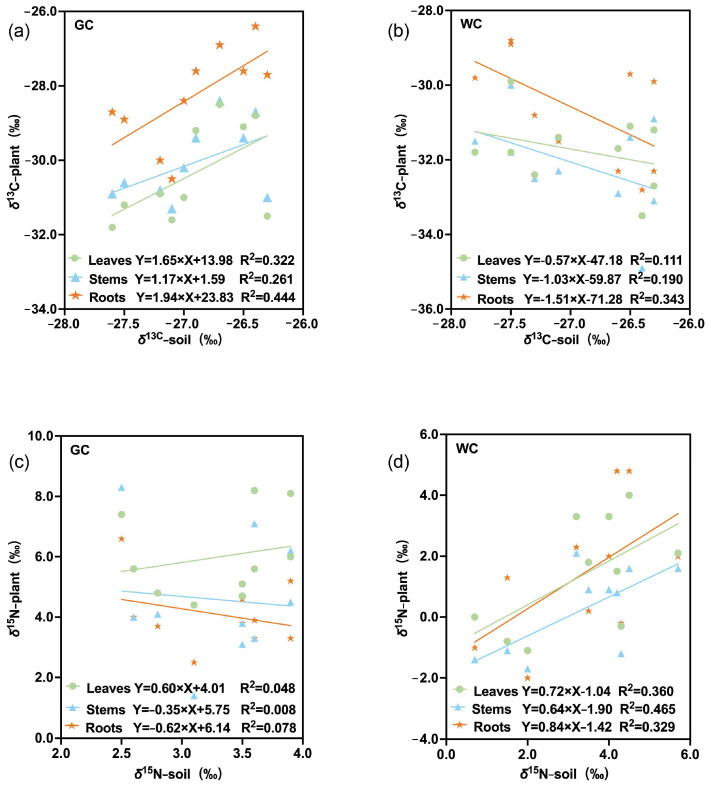
*δ*^13^C and *δ*^15^N correlations between soil and plant tissues under GC and WC cultivation conditions. (**a**) *δ*^13^C between soil and plant tissues under GC conditions; (**b**) *δ*^13^C between soil and plant tissues under WC conditions; (**c**) *δ*^15^N between soil and plant tissues under GC conditions; (**d**) *δ*^15^N between soil and plant tissues under WC conditions.

Furthermore, *δ*^13^C and *δ*^15^N correlations between soil and tissue type were also explored and showed obvious differences under GC and WC conditions (Figure 2a–d). GC soil *δ*^13^C values were found to have strong positive correlations with GC plant tissues (*r* > 0.511, *p* < 0.05), with positive linear correlations showing a good fit for above-ground and below-ground plant tissues (Figure 2a). The correlations were significantly lower for WC soils and plants (Figure 2b), with negative correlations suggesting limited isotopic influence from the soil. Conversely, there was a strong positive correlation for *δ*^15^N between WC soil, leaves, stems and roots (*r* > 0.573, *p* < 0.05), with the stems showing the highest plant tissue R^2^ value (R^2^ = 0.465), followed by leaves and then roots. However, the linear regression analyses revealed extremely weak correlations between all plant tissues and soil *δ*^15^N under GC conditions, with all R^2^ values approaching 0, indicating almost no linear association between soil *δ*^15^N and tissue *δ*^15^N.

### 2.4. Characterization of Metals in T. hemsleyanum and Soils

Soils serve as the primary source of nutrients for plant growth and may also contain various geogenic and anthropogenic metal elements. The study investigated the accumulation of metals—specifically chromium (Cr), nickel (Ni), copper (Cu), arsenic (As), cadmium (Cd), and lead (Pb)—in different plant tissues (roots, stems, and leaves) and corresponding soils under the two cultivation systems (Table 3).

In this study, both cultivation systems used the same starting soil, sheep organic fertilizer and ash additives. For intra-system tissue comparisons, the concentrations of three metals (Cr, Cu, and As) were significantly higher in roots than in leaves and stems under the GC regime, with the most pronounced differences observed for Cr. In contrast, Cd, Ni, and Pb concentrations were comparable across the three plant tissues. Under the WC regime, Cr and As concentrations were marginally higher in stems and roots than in leaves; Cu concentrations were more elevated in roots relative to leaves and stems, while no significant differences in Ni, Cd, and Pb concentrations were detected among the three plant tissues.

For inter-system comparisons between GC and WC, root tissues showed a significant difference for Pb and Cd concentrations. The values of Pb and Cd in WC roots (837.6 ± 720.3 µg/kg and 333.8 ± 130.7 µg/kg, respectively) were much higher than those in GC roots (522.4 ± 124.4 µg/kg and 149.6 ± 24.7 µg/kg, respectively). The mean concentrations of the remaining metals in roots were comparable between the two cultivation systems, at approximately 6.1 mg/kg for Cr, 5.5 mg/kg for Ni, 8.7 mg/kg for Cu, and 110.1 µg/kg for As. WC stem tissues had significantly higher Cr (3.2 mg/kg) and Pb (1775.4 µg/kg) values than GC stems (1.7 mg/kg and 493.0 µg/kg, respectively). Mean leaf concentrations of Cr (0.8 mg/kg), Cu (5.3 mg/kg), Ni (2.4 mg/kg), As (41 µg/kg), and Cd (200 µg/kg) were similar across the two cultivation systems; however WC leaves accumulated significantly more Pb (1122.1 μg/kg) than GC leaves (352.4 μg/kg). Overall, the highest metal concentrations were found in the roots, as they are directly able to absorb and store metals from the soil, although the source of high Pb concentrations in WC leaves is most likely due to atmospheric deposition from anthropogenic sources.

A comparison of soil metal distributions between GC and WC systems showed that Cd concentrations had no significant change between cultivation systems (*p* > 0.05), while Pb showed highly significant differences (*p* < 0.01) across the two cultivation systems due to anthropogenic input. Overall, the results show that Cr, Ni, Cu and As metal migration from soil to plant is primarily affected by cultivation environment, rather than external factors [33].

Although there were significant differences between the metal contents of the two cultivation soils, most plant tissue metal concentrations (Cd, Cr, Ni, Cu, As) were very similar across both cultivation methods. This indicates that soil metal concentration had a limited effect on the uptake ability, absorption, accumulation, and translocation into plant tissues regardless of the cultivation method. BCF and TF, which are typically used to demonstrate metal phytoremediation [34], were used to evaluate these capacities, and the results are presented in Table 4.

Cd is evenly distributed among plant tissues and soils in this study; however, this metal exhibited BCF values > 1 in all tissues under WC conditions, and root tissues showed significantly higher values in WC than in GC. Cd is not an essential element for plant physiology and is derived from anthropogenic activities, rather than being naturally present in soils. However, it is highly mobile, bio-available, and easily taken up by plants [15,35]. Cd TF values were >2, demonstrating a high capacity for Cd uptake from soil and subsequent translocation from the roots to above-ground tissues.

Pb is a widely distributed metal worldwide. It has an average concentration of 20 mg/kg in the Earth’s crust and is mainly concentrated in ore deposits. It is ultimately released into living organisms through anthropogenic sources such as vehicle emissions, coal combustion, and lead-based paints, among others [36]. Higher values in WC soil and plant tissue samples suggest that lead atmospheric deposition has occurred, affecting WC plant tissue and soil Pb distributions more than the greenhouse-protected GC soil and plants [37]. Although Pb concentrations in both WC and GC soils are found to be relatively high, plant tissue Pb contents are within acceptable food safety limits set by the FAO/WHO [38]. Plant tissue Pb BCF data for both cultivation systems show values between 0.013 and 0.035, suggesting that *T. hemsleyanum* has a low Pb uptake ability from soil. Nevertheless, significantly higher Pb concentrations were found in WC soils and plant tissues than in corresponding GC counterparts, clearly indicating that atmospheric deposition of Pb may play a dominant accumulation role in natural forest and open farmland ecosystems in this region.

The remaining metals (As, Cr, Cu, Ni) had lower BCFs (<0.6) and similar TF values (about 1.0), demonstrating that *T. hemsleyanum* does not strongly bioaccumulate these metals from the soil, but some metal transfer occurs from roots to above-ground tissues. In summary, Cd and Pb exhibited the highest concentrations in plant tissues compared to their soils, indicating these elements are most prone to bioaccumulation in *T. hemsleyanum* tissues.

### 2.5. Effects of Soil–Plant Metals on Stable Isotopes

**Figure 3:**
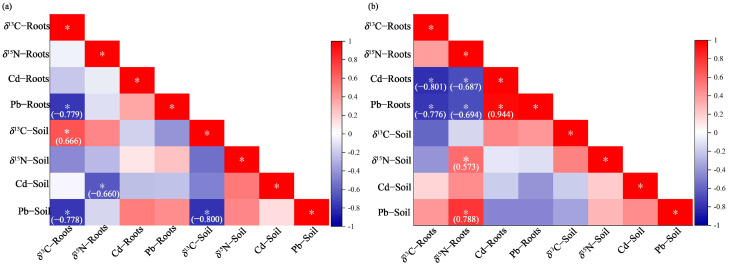
Correlations between soil, roots, metals and stable isotopes from two cultivation systems (**a**) GC; (**b**) WC. Note: significant correlations are marked with * (*p* < 0.05).

Cd and Pb exhibited significant differences between the two cultivation methods, so they were selected as parameters to investigate their impact on C and N cycling in *T. hemsleyanum* [18,39]. Correlation matrices were generated, enabling a comparative assessment of how cultivation type influenced metal-isotope linkages (Figure 3). While Pb and Cd soil concentrations were within the regulatory limits (Cd < 0.6 ppm and Pb < 170 ppm [40]) under both cultivation methods, they showed significant differences in plant tissues. Soil Pb and Cd concentrations showed no correlation with roots for either production system that could be solely explained by uptake in root tissues [41]. Under GC conditions, strong negative correlations were seen for soil Pb with *δ*^13^C root and soil values (*r* ≈ −0.8, *p* < 0.05), root Pb and *δ*^13^C root values (*r* = −0.779, *p* < 0.05) and soil Cd and *δ*^15^N root values (*r* ≈ −0.7, *p* < 0.05).

Under WC production, soil Pb had a strong positive correlation with *δ*^15^N root values (*r* = 0.788, *p* < 0.05). However, root Cd and Pb showed negative correlations with *δ*^13^C and *δ*^15^N root values (*p* < 0.05), although correlation coefficients were slightly lower for *δ*^13^C (*r* ≈ −0.8) than for *δ*^15^N (*r* ≈ −0.7). In this study, GC represents a closed system, and more soil C was retained than in WC, although different Pb and Cd concentrations may also affect the carbon dynamics [42]. Previous research has shown that increased soil metal stress results in lower plant *δ*^13^C values, resulting in photosynthetic efficiency reductions related to ribulose-1,5-bisphosphate (RuBP) carboxylase [43].

In this study, WC can be classified as an open cultivation system and is impacted more by external anthropogenic metal sources than GC, which results in a higher positive Cd and Pb correlation between soil and roots [44]. Hence, the contrasting metal-isotope relationships between GC and WC suggest that closed-system cultivation buffers metal impacts on nutrient dynamics, and subsequent internal tissue transfer is more influenced by root metal concentrations than by bulk environmental pools.

## 3. Materials and Methods

### 3.1. Experimental Design and Sampling

The experimental site was located in Wuyi County, Jinhua City, Zhejiang Province, in east China (28°31′ ~ 29°03′ N and 119°27′ ~ 119°58′ E). The climate is mid-subtropical monsoon, with four distinct seasons, mild temperatures and humidity, with an annual rainfall of approximately 1600 mm.

Two *T. hemsleyanum* production systems were used—“greenhouse cultivation (GC)” and “simulated-wild cultivation (WC)”. WC plants were grown in pots in hilly, mountainous regions, where the temperature ranges from 0 to 15 °C to simulate the natural growing conditions of wild plants. Cuttings were placed in 30 × 30 × 30 cm pots in the understory of trees, and organic sheep manure was added at planting. Ash, recovered from burning plant wastes (weeds, rice straw or other crops), was added after 1 year. The WC plants received only rainwater and some surface water expressed from mountain springs that could naturally seep into the pots. GC plants were grown along the valley floor on agricultural land in shaded greenhouses covered with plastic. Cuttings were planted in pots sized similar to those used for WC plants. Mean daily temperatures ranged from 10 to 20 °C, and the plants were irrigated several times per week with local groundwater. GC plants received similar organic fertilizer treatments as the WC plants (sheep manure and wood ash) at planting and after 1 year.

**Figure 4:**
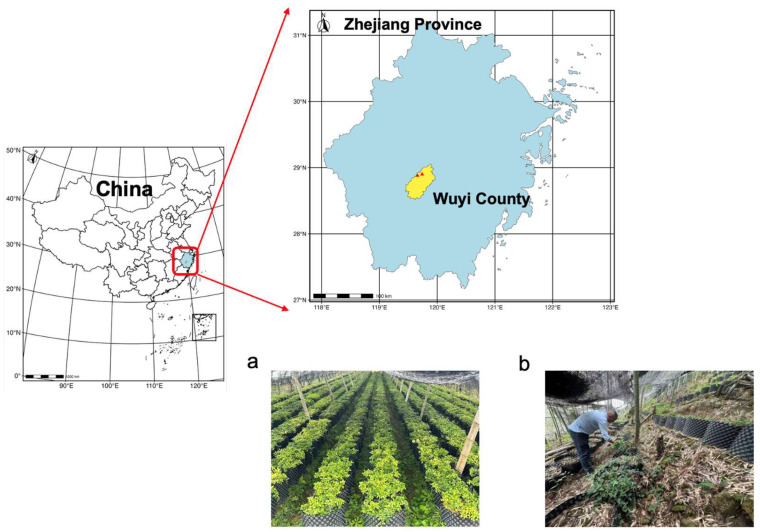
Location and set-up of the two cultivation sites for *Tetrastigma hemsleyanum* in Wuyi County, Zhejiang Province, East China. (**a**) Greenhouse cultivation method; (**b**) simulated-wild cultivation method.

All samples were planted between April and May 2020 at two adjacent farms, using both GC (Figure 4a) and WC (Figure 4b) growing systems. The soil used in both GC and WC pots was sourced from the same field at each farm. The mature plants were harvested between November 2022 and January 2023. There were 30 pots per cultivation method at each farm, and soil and plant tissues were sampled and pooled from 3 pots to make a single composite sample. Hence, 60 tissue samples (including leaves, stems, and roots) and 20 corresponding soil samples were collected from two cultivation systems at each farm.

Freshly harvested plants were washed with deionized water to remove soil and debris, then separated into various tissue parts—leaves, stems, and roots—and chopped into smaller pieces for drying. Plant tissues and soil samples were freeze-dried for 48 h, ground, and passed through a 100-mesh sieve to obtain homogeneous powders for subsequent analysis. The soil samples were air-dried at room temperature, ground, and then sieved through a 1 mm mesh prior to analysis.

### 3.2. Stable Isotope Analysis

Carbon and nitrogen isotopes were analyzed following Nie et al. [45] using a Vario Isotope Cube elemental analyzer (Elementar, Langen, Germany) coupled to an isotope ratio mass spectrometer (Elementar, Langen, Germany). For carbon and nitrogen isotope analysis, approximately 5.0 mg of ground plant tissue and 20.0 mg of soil were weighed into 6 × 9 mm tin boats, which were then combusted in a WO_3_ reactor at 1150 °C. The combustion gases were reduced to CO_2_ and N_2_ at 850 °C, separated using a CentrION diluter, and introduced into an isotope ratio mass spectrometer for simultaneous quantification of both gases.

Hydrogen and oxygen isotopes were pyrolyzed in a Pyro Cube (Elementar, Langen, Germany). The plant samples (0.5 mg) were sealed in 4 × 6 mm silver boats and pyrolyzed at 1450 °C in a carbon-glass reactor to generate H_2_ and CO, which were then analyzed using an Isoprime 100 Isotope Ratio Mass Spectrometer (Isoprime, Manchester, UK). Prior to H and O analysis, the samples and reference materials were freeze-dried at −60 °C for three days to remove all exchangeable water and subsequently equilibrated for five days in the laboratory exposed to local atmospheric conditions [46].

Stable isotope ratios were calculated using Equation (1):

*δE* = (*R*_sample_ − *R*_reference_)/*R*_reference_ (1)

where *E* is the isotope of the target element, *R_sample_* is the abundance ratio of heavy and light isotopes in the measured samples (i.e., ^13^C/^12^C, ^15^N/^14^N, ^18^O/^16^O, and ^2^H/^1^H) and *R_reference_* is the abundance ratio of heavy and light isotopes in international reference materials. The isotope values are reported relative to known international reference materials, where *δ*^13^C is V-PDB, *δ*^15^N is air, and *δ*^18^O and *δ*^2^H are standard mean ocean water (SMOW). A tuberous root sample was used as a quality control standard, and the analytical precision was lower than 0.1‰ for *δ*^13^C, 0.1‰ for *δ*^15^N, 3‰ for *δ*^2^H and 0.5‰ for *δ*^18^O, respectively.

Multipoint calibration was performed using commercial stable isotope reference materials, which included BCR657 (*δ*^13^C = −10.76‰), IAEA-N-2 (*δ*^14^N = 20.3‰), B2155 (*δ*^13^C = −26.98‰, *δ*^14^N = 5.94‰), USGS40 (*δ*^15^N = −4.52‰), USGS54 (*δ*^2^H = −150.4‰, *δ*^18^O = 17.79‰), USGS55 (*δ*^2^H = −28.2‰, *δ*^18^O_V-SMOW_ = 19.12‰), USGS64 (*δ*^13^C_V-PDB_ = −40.81‰), and USGS56 (*δ*^2^H_V-SMOW_ = −44.0‰, *δ*^18^O_V-SMOW_ = 27.23‰).

### 3.3. Elemental Analysis

Multi-element analysis was performed following Mei et al. [47]. For tissue samples, approximately 0.1 g of dried powder was weighed and digested with 7.0 mL of nitric acid (HNO_3_, ≥65%, ppb grade, CNW Technologies, Shanghai, China). For soil samples, approximately 0.1 g of dried powdered soil was weighed and digested with 7.0 mL of nitric acid and 0.2 mL of hydrofluoric acid (HF, 48–50%, for trace metal analysis, <1 ppb, CNW Technologies, Shanghai, China). The acidified solutions of *T. hemsleyanum* and soil were placed in a TOPEX+ digestor (PreeKem, Shanghai, China) and heated to 200 °C for 30 min. Then, 1.0 mL of perchloric acid (70–72 wt%, Tianjin Xinyuan Chemical Company Ltd., Tianjin, China) was added to the digested soil solution after cooling, and the solutions were transferred to a graphite heating block (GT-400, PreeKem, Shanghai, China) at 200 °C for acid reduction until the volume of the soil solution was approximately 0.5 mL. For plant samples, the solutions were transferred to a heating block at 180 °C for acid reduction until yellow acid fumes were exhausted. The plant and soil digestion solutions were diluted to 50 mL with deionized water. Then, 5 mL of each diluted soil solution was further diluted with deionized water to 25 mL, after which all samples were filtered and analyzed.

An inductively coupled plasma mass spectrometer (ICP-MS, 8900 triple quadrupole, Agilent Technologies, Santa Clara, CA, USA) was employed to determine metal concentrations in plant and soil samples using the following parameters: Rf power was set at 1550 W, plasma gas (argon, purity greater than 99.99%) flow rate of 15 L/min, MicroMist nebulizer gas was set at 1.05 L/min, and temperature of 2 °C in a Scott-type spray chamber. Cr, Cu, Ni, Cd, and Pb metals were determined using kinetic energy discrimination (KED), with a mass-to-charge ratio of 51, 63, 60, 111, and 208, respectively. As was determined in mass-shift mode using O_2_ cell gas, with a mass-to-charge ratio of 75 to 91. An internal standard containing 50.0 μg·L^−1^ of Ge (GSB 04-1728-2004), Rh (GSB 04-1746-2004), and Re (GSB 04-1745-2004) (CNRM, Beijing, China) was used to monitor instrument drift. Certified reference materials (Soil-GBW07403a and Ginger-GBW10202) were obtained from the National Sharing Platform for Reference Materials (CNRM, Beijing, China) and used as laboratory quality control samples. Analyzed metal recoveries ranged between 85 and 110%.

### 3.4. Calculation of Stable Isotope Fractionation

Fractionation factors (α) are sensitive indicators of isotopic discrimination processes occurring within the plant and between the plant and its environment [48]. The isotope fractionation coefficient (*α*) between roots and other plant tissues was calculated using Equation (2) [49]:

*α* = (*δ*E_1_ + 1000)/(*δ*E_2_ + 1000) (2)

where *δ*E_1_ and *δ*E_2_ represent the isotope values in the upper and lower parts of *T. hemsleyanum*, respectively, and E refers to the roots, stems and leaves of the plant.

### 3.5. Calculation of Metal Bioconcentration and Translocation Factors

The efficiency of metal uptake from the soil under WC and GC cultivation practices was calculated for roots, stems, and leaves using a bioconcentration factor (BCF) in Equation (3) [50]:

(3) $$\mathrm{BCF}=\frac{Metal\;concentration\;in\;tissue}{Metal\;concentration\;in\;soil}$$

where BCF values exceeding 1 reflect effective metal accumulation by plants, while values below 1 imply a restricted uptake.

The translocation factor (TF) is defined as the ratio of the metal concentration in shoots to that in roots [28] and is calculated for WC and GC cultivation practices using Equation (4):

(4) $$\mathrm{TF}=\frac{\left(Metal\;concentration\;in\;leaves+Metal\;concentration\;in\;stems\right)}{Metal\;concentration\;in\;roots}$$

### 3.6. Data Statistics and Visualization

One-way analysis of variance (ANOVA), followed by Tukey’s test (*p* < 0.05) for *T. hemsleyanum* tissues, and *t*-tests between the two growing conditions were conducted using SPSS 18.0 software (International Business Machines Corporation, Armonk, NY, USA) to determine significant differences. Visualizations were conducted in GraphPad Prism 10.4.0 (GraphPad Software, Boston, MA, USA).

## 4. Conclusions

This study explores stable isotopes and metal contents of leaves, stems, tuberous roots, and soils under two different *T. hemsleyanum* cultivation systems (GC and WC). The results show that *δ*^13^C and *δ*^15^N values were significantly more positive under GC methods than in WC. GC systems indicated more rapid carbon fixation and metabolism, and faster nitrogen turnover due to comparatively warmer growing conditions and increased water availability than WC, whereas WC conditions resulted in slower nitrogen cycling and uptake. While soil *δ*^13^C and *δ*^15^N values did not differ significantly between the two systems, soil–plant isotope correlations displayed contrasting isotopic uptake patterns for GC and WC systems.

Most metals showed low tissue bioconcentration with no obvious hyperaccumulation tendency, and the cultivation method had only limited influence on plant metal uptake and tissue translocation; however, Pb and Cd concentrations were higher in roots and leaves. Further investigation showed that these two metals are clearly correlated with C and N cycling in soil–plant systems, and that GC systems buffer metal impacts on overall carbon dynamics, while GC plants are more prone to soil–root metal accumulations than those from externally available environmental pools used by WC.

These findings provide a reliable technique to authenticate *T. hemsleyanum* cultivated using wild and simulated-wild production systems from their greenhouse-grown counterparts. It not only offers a new tool for the industry to prevent market fraud and mislabeling issues, but also establishes a framework for authenticating other ‘agro-ecological’ labeled products. While the single geographic locality scope may present possible experimental limitations, future work will expand these findings for multi-region validation, introduce multi-omics to evidence plant regulatory mechanisms, conduct longer-term monitoring to improve traceability and more robustly standardize cultivation characteristics of *T. hemsleyanum*.

## Figures and Tables

**Table 1:** Stable isotope distributions and fractionation coefficients of tissues under the two cultivation systems.

Cultivation System		*δ*^13^C (‰)			*δ*^15^N (‰)			*δ*^2^H (‰)			*δ*^18^O (‰)		
		Mean	SD	*α*	Mean	SD	*α*	Mean	SD	*α*	Mean	SD	*α*
	Roots	−28.3 ^a^ **	1.3	-	4.1 ^b^ **	1.2	-	−13 ^a^	5	-	27.1 ^a^	0.9	-
GC	Stems	−30.1 ^b^ **	1.0	0.9981	4.6 ^ab^ ***	2.1	1.0005	−59 ^b^ **	4	0.9534	20.9 ^b^	0.8	0.9940
	Leaves	−30.4 ^b^ *	1.3	0.9997	6.0 ^a^ ***	1.4	1.0014	−65 ^c^	7	0.9936	26.7 ^a^	1.8	1.0057
	Roots	−30.7 ^a^ **	1.5	-	1.4 ^a^ **	2.3	-	−12 ^a^	5	-	26.4 ^a^	0.9	-
WC	Stems	−32.1 ^b^ **	1.4	0.9986	0.3 ^a^ ***	1.4	0.9989	−50 ^b^ **	6	0.9615	21.0 ^b^	0.8	0.9947
	Leaves	−31.8 ^ab^ *	1.0	1.0003	1.4 ^a^ ***	1.8	1.0011	−62 ^c^	10	0.9874	27.2 ^a^	0.6	1.0061

Note: Superscript letters (^a^, ^b^, ^c^) indicate significant differences among tissues within the same cultivation system, and asterisks denote significant differences between cultivation systems (*, *p* < 0.05; **, *p* < 0.01; ***, *p* < 0.001); *α* is the stable isotope fractionation coefficient between the roots and stems or leaves.

**Table 2:** Stable isotopes of greenhouse cultivation (GC) and simulated-wild cultivation (WC) soils.

Variable	GC				WC				*p* Value
	Max	Min	Mean	SD	Max	Min	Mean	SD	
C (%)	9.5	1.2	3.4	2.5	2.8	1.4	2.2	0.7	0.019
N (%)	0.7	0.1	0.3	0.2	0.3	0.1	0.2	0.1	0.031
*δ*^13^C (‰)	−26.3	−27.6	−26.9	0.5	−26.3	−27.8	−26.9	0.6	0.885
*δ*^15^N (‰)	3.9	2.5	3.3	0.5	5.7	0.7	3.4	1.5	0.858

Note: Significant differences are observed at *p* < 0.05.

**Table 3:** Metal contents of different *T. hemsleyanum* tissues and soil under different cultivation methods (values are based on dry weight).

Cultivation System	Tissue/Soil	Cr	Ni	Cu	As	Cd	Pb
		mg/kg	mg/kg	mg/kg	μg/kg	μg/kg	μg/kg
Greenhouse	Roots	7.5 ± 8.1 ^a^	3.4 ± 0.6 ^a^	9.5 ± 2.8 ^a^	103.1 ± 26.8 ^a^	149.6 ± 24.7 ^a^ *	522.4 ± 124.4 ^a^ **
	Stems	1.7 ± 0.7 ^b^ *	1.5 ± 0.5 ^a^	6.9 ± 1.6 ^b^	65.6 ± 35.2 ^b^	291.3 ± 212.9 ^a^	493.0 ± 182.8 ^a^ **
	Leaves	0.9 ± 0.5 ^b^	1.7 ± 0.8 ^a^	5.9 ± 1.3 ^b^	47.2 ± 31.7 ^b^	169.3 ± 159.8 ^a^	352.4 ± 207.1 ^a^ *
	Soil	26.8 ± 8.7 *	8.2 ± 1.6 *	21.9 ± 10.4 *	9044.6 ± 3196.2 *	316.4 ± 41.0	36448.5 ± 5099.2 **
Simulated-Wild	Roots	4.7 ± 2.5 ^a^	5.8 ± 5.0 ^a^	7.8 ± 2.0 ^a^	121.9 ± 39.4 ^a^	333.8 ± 130.7 ^a^ *	1385.6 ± 397.7 ^a^ **
	Stems	3.2 ± 1.5 ^a^ *	2.7 ± 2.1 ^a^	5.9 ± 1.5 ^b^	87.1 ± 41.2 ^a^	511.7 ± 345.1 ^a^	1775.4 ± 941.1 ^a^ **
	Leaves	0.7 ± 0.4 ^b^	2.9 ± 1.9 ^a^	4.6 ± 0.9 ^b^	34.5 ± 14.8 ^b^	231.0 ± 176.7 ^a^	837.6 ± 720.3 ^a^ *
	Soil	17.9 ± 6.3 *	6.0 ± 1.7 *	13.2 ± 4.7 *	5296.0 ± 1018.9 *	301.1 ± 70.1	51592.8 ± 4635.3 **

Note: Values are shown as mean ± standard deviation. Superscript letters (^a^, ^b^) indicate significant differences among tissues within the same cultivation system, and asterisks denote significant differences between cultivation systems (*, *p* < 0.05; **, *p* < 0.01).

**Table 4:** Bioconcentration factor (BCF) and translocation factor (TF) values of metals in plant tissues under greenhouse cultivation (GC) and simulated-wild cultivation (WC) conditions.

Cultivation	Metals	BCF			TF
		Leaves	Stems	Roots	
Greenhouse	Cr	0.050	0.072 *	0.203	0.800
	Ni	0.313	0.247	0.505	1.122
	Cu	0.352	0.399	0.571	1.500
	As	0.005	0.008	0.012	1.173
	Cd	0.559	1.382	0.509 *	2.851
	Pb	0.013	0.017	0.014 *	1.957
Simulated-Wild	Cr	0.044	0.212 *	0.289	1.066
	Ni	0.574	0.577	1.051	1.443
	Cu	0.412	0.504	0.691	1.384
	As	0.007	0.017	0.024	1.017
	Cd	1.094	1.834	1.196 *	2.324
	Pb	0.023	0.035	0.027 *	2.101

Note: Asterisks denote significant differences between GC and WC conditions (*, *p* < 0.05).

## Data Availability

The data presented in this study are available on request from the corresponding author.
